# Epigenetic Modifications Associated to Neuroinflammation and Neuropathic Pain After Neural Trauma

**DOI:** 10.3389/fncel.2018.00158

**Published:** 2018-06-07

**Authors:** Clara Penas, Xavier Navarro

**Affiliations:** ^1^Institut de Neurociències, Departament de Biologia Cellular, Fisiologia i Immunologia, Universitat Autònoma de Barcelona, Barcelona, Spain; ^2^Centro de Investigación Biomédica en Red sobre Enfermedades Neurodegenerativas, Madrid, Spain

**Keywords:** traumatic injury, inflammation, neuronal hyperexcitability, neuropathic pain, epigenetic enzymes

## Abstract

Accumulating evidence suggests that epigenetic alterations lie behind the induction and maintenance of neuropathic pain. Neuropathic pain is usually a chronic condition caused by a lesion, or pathological change, within the nervous system. Neuropathic pain appears frequently after nerve and spinal cord injuries or diseases, producing a debilitation of the patient and a decrease of the quality of life. At the cellular level, neuropathic pain is the result of neuronal plasticity shaped by an increase in the sensitivity and excitability of sensory neurons of the central and peripheral nervous system. One of the mechanisms thought to contribute to hyperexcitability and therefore to the ontogeny of neuropathic pain is the altered expression, trafficking, and functioning of receptors and ion channels expressed by primary sensory neurons. Besides, neuronal and glial cells, such as microglia and astrocytes, together with blood borne macrophages, play a critical role in the induction and maintenance of neuropathic pain by releasing powerful neuromodulators such as pro-inflammatory cytokines and chemokines, which enhance neuronal excitability. Altered gene expression of neuronal receptors, ion channels, and pro-inflammatory cytokines and chemokines, have been associated to epigenetic adaptations of the injured tissue. Within this review, we discuss the involvement of these epigenetic changes, including histone modifications, DNA methylation, non-coding RNAs, and alteration of chromatin modifiers, that have been shown to trigger modification of nociception after neural lesions. In particular, the function on these processes of EZH2, JMJD3, MeCP2, several histone deacetylases (HDACs) and histone acetyl transferases (HATs), G9a, DNMT, REST and diverse non-coding RNAs, are described. Despite the effort on developing new therapies, current treatments have only produced limited relief of this pain in a portion of patients. Thus, the present review aims to contribute to find novel targets for chronic neuropathic pain treatment.

## Introduction

Neuropathic pain appears as a consequence of a lesion or disease affecting the CNS or PNS (Freynhagen and Bennett, 2009). It is estimated that around 6–8% of general population suffers of chronic pain with neuropathic characteristics (Torrance et al., 2006; Bouhassira et al., 2008). Neuropathic pain is characterized by presenting pain under non-painful stimulus (allodynia), increased pain after painful stimulus (hyperalgesia) and spontaneous pain without stimuli. Neuropathic pain is considered the result of neural plasticity, produced by both an increase in the sensitivity and excitability of primary sensory neurons in PNS, and an increase in the activity and excitability of nociceptive neurons in the spinal cord and the brain (Woolf and Salter, 2000; Julius and Basbaum, 2001). Thus, injury to the PNS or CNS induces maladaptive changes in neurons along the nociceptive pathway that can cause neuropathic pain. The cellular and molecular alterations underlying such pain have been described at different locations within the nociceptive pathways. So far, changes have been reported in the peripheral nerve, the DRG, the dorsal horn, the brainstem, particularly in the nucleus raphe magnus, the thalamic relay nuclei, and the brain cortex (Geranton and Tochiki, 2015). Despite the nature of the neural traumatic injury, there is an alteration of similar structures triggering peripheral and central sensitization. Several mice models of human neuropathic pain have been developed to try to understand these processes (Colleoni and Sacerdote, 2010). Despite the effort on developing new therapies, current treatments have only produced limited relief of this pain in a portion of patients (Dworkin et al., 2003; Costigan et al., 2009) and there is a significant proportion of patients showing resistance to medication. For example, a reduced responsiveness to opioid analgesics is typically seen in patients with neuropathic pain (Campbell and Meyer, 2006).

Spinal cord glial cells, microglia and astrocytes, together with blood borne macrophages, play a critical role in the induction and maintenance of neuropathic pain by releasing powerful neuromodulators such as pro-inflammatory cytokines and chemokines (McMahon et al., 2005). Cytokines and chemokines regulate synaptic transmission and plasticity (Gao and Ji, 2010), and enhance neuronal excitability after neural lesions. Inflammation, and the neural injury *per se*, contribute to hyperexcitability and therefore to the development of neuropathic pain, inducing the altered expression, trafficking and functioning of ion channels expressed by sensitized sensory neurons (Hains and Waxman, 2007; Dib-Hajj et al., 2009; Tibbs et al., 2016). Then, positive feedback loops between enhanced electrical activity of neurons and combined activation of peripheral and central inflammatory cells help to sustain neuroinflammation and chronic neuropathic pain (Miller et al., 2009; Xie et al., 2009; Walters, 2012). The pathophysiological changes that underlie the generation and maintenance of neuropathic pain could be summarized in two sections. On one side, the induction of pro-inflammatory neuromodulator expression, mainly released by glial cells and macrophages. On the other side, the altered expression of channels, receptors, transporters and neurotransmitters by neuronal cells.

There is strong evidence that the molecular changes developing pain states after traumatic injuries of the nervous system, are governed by epigenetic mechanisms. Epigenetic mechanisms are inherited and reversible modifications to nucelotides or chromosomes that alter gene expression without changing DNA sequence. Epigenetic mechanisms are capable to sustain the long-lasting effects on gene activity in response to environmental stimuli, observed in neuropathic pain. Epigenetic mechanisms are alterations that produce changes in gene expression that occur without alteration in DNA sequence. These non-genetic alterations are regulated by two major epigenetic modifications: chemical modifications of DNA (DNA methylation) and covalent modification of histones associated with DNA (histone modifications). These alterations change the chromatin state between euchromatin or heterochromatin, which are transcriptionally accessible/active, or inaccessible/inactive states of chromatin, respectively. More recently, a third system included is non-coding RNA (ncRNA)-associated gene silencing and microRNA alteration. DNA methylation, produced by DNMTs, is linked to transcriptional silencing. It produces gene repression by physically impeding the binding of transcriptional proteins to the gene and because methylated DNA may be bound by proteins can modify histones, thereby forming heterochromatin. Methylation of histones can either increase or decrease transcription of genes, depending on which amino acids in the histones are methylated, and how many methyl groups are attached. Methylation events that weaken chemical attractions between histone tails and DNA increase transcription, because they enable the DNA to uncoil from nucleosomes, and thus forming euchromatin. Histone acetylation in lysine residues is promoted by HATs, and deacetylation by HDACs. The lysine residues have a positive charge that binds tightly to the negatively charged DNA and form closed chromatin structure, inaccessible to transcription factors (Jenuwein and Allis, 2001; Ruthenburg et al., 2007). Thus, acetylation removes positive charges of histones, opens the condensed chromatin structure, and allows transcriptional machines easier access to promoter regions. Finally, histone methylation is associated with either transcriptional activation, inactivation, or silent genomic regions (Jenuwein and Allis, 2001; Consortium et al., 2007). A summary of epigenetic marks associated to transcriptional activation or gene inactivation or silencing is shown in **Table 1**. Besides, non-coding RNAs play also an important role. From these, microRNAs contribute to inhibit translation and/or degradation of mRNAs (Grishok et al., 2001; Bartel, 2004; He and Hannon, 2004).

**Table 1:** Major epigenetic marks of active and repressive gene expression.

Epigenetic markers	Euchromatin (active expression)	Heterochromatin (repressed expression)
DNA methylation	Hypomethylation	Hypermethylation
Histone methylation	H3K4me2/3, H3K9me	H3K27 me2/3, H3K9me2/3
Histone acetylation	Hypercetylated H3 and H4	Hypoacetylated H3 and H4

Within this review, we describe the major epigenetic enzyme alterations observed after traumatic neural injuries that affect the expression of pro-inflammatory neuromodulators, and intrinsic neuronal excitability, promoting neuropathic pain. Although the importance of epigenetics in traumatic injuries is becoming evident, the discoveries in this field are still limited, and further research needs to be done to clarify the molecular pathways underlying these events. That we are at this initial point is clearly evident when in the literature there is no agreement, and certain contradictory findings are described. The present review tries to shed light onto these mechanisms that may be useful to develop therapeutic interventions for reducing neuropathic pain.

## Induction of Pro-Inflammatory Neuromodulator Expression

**FIGURE 1:**
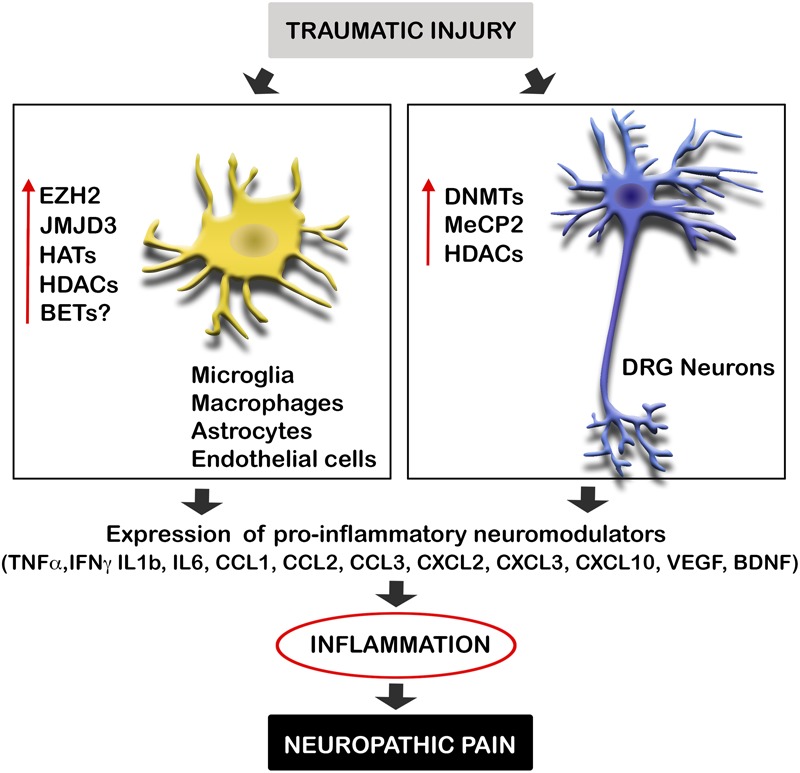
Traumatic injuries of the nervous system induce epigenetic alterations triggering inflammation and neuropathic pain. Neural injuries produce an increase of the expression of several epigenetic enzyme in microglia, macrophages, astrocytes, endothelial cells, and neurons of the DRG after neural injuries. These enzymes alter the promoter state of several pro-inflammatory neuromodulators inducing their expression. Enhanced gene expression of these cytokines, chemokines, and neurotrophic factors, produce inflammation, and consequent neuropathic pain. BET, bromodomain and extra terminal domain; DNMTs, DNA methyltransferases; EZH2, enhancer of zeste 2 polycomb repressive complex 2 subunit; HAT, histone acetyltransferase enzymes; HDAC, histone deacetylases; JMJD3, jumonji domain containing 3; MeCP2, methyl-CpG-binding protein 2.

Diverse causes of neuropathic pain are associated with excessive inflammation in both the PNS and CNS, which may contribute to the initiation and maintenance of persistent pain (Ellis and Bennett, 2013). Inflammation is produced by the release of neuromodulators, including cytokines, chemokines, vasoactive peptides, lipid mediators, and proteolytic enzymes. Such chemical mediators produce the sensitization of central neurons and nociceptors, and are released by both resident cells (microglia, astrocytes, Schwann cells) and infiltrating cells (neutrophils, macrophages). Several studies suggest that after traumatic injuries, the expression of these mediators is altered by the dysregulation of epigenetic enzymes (**Figure 1**). These enzymes are methyltransferases and demethylases, as well as HDACs and HATs, which alter the promoter state of these neuromodulators. Therefore, epigenetic enzymes contribute to create chemical mediator promoter accessibility to the transcription machinery, enhancing gene expression. Inhibition of these enzymes has been described to produce a decrease of inflammation as well as neuropathic pain.

Besides, microRNAs play also an important role on the generation of neuropathic pain. Genome or micro-array data of several investigations report that microRNAs are differentially regulated in neuropathic pain models in DRG neurons, sciatic nerve, spinal cord and nucleus accumbens (Imai et al., 2011; Kusuda et al., 2011; Genda et al., 2013; Bali et al., 2014; Chang et al., 2017). These non-coding RNAs seem to affect both inflammation and excitability after neural trauma.

### Histone and DNA Methylation

After traumatic injury, there is altered expression of the histone methyltransferase EZH2, the histone demethylase JMJD3 or KDM6b and the transcriptional repressor MeCP2. EZH2 and JMJD3 have opposite actions on the H3K27me2/3, which is a repressive biomarker that induces chromatin compaction and thus gene inactivation. However, literature reports indicate that both enzymes have an inflammatory action causing neuropathic pain. Thus, intriguing molecular events onto the cross-talk between opposite enzymes need to be clarified.

#### EZH2

EZH2, is a subunit of the polycomb repressive complex 2 (PRC2), and catalyzes the di- and tri-methylation of histone H3 on lysine 27 (H3K27Me2/3), resulting in gene silencing. Although increases of PRC2 complex may contribute to protect neurons against neurodegeneration (von Schimmelmann et al., 2016), very recently, a couple of investigations indicate that the methyltransferase EZH2 is involved in the generation and maintenance of neuropathic pain by inflammatory mechanisms.

EZH2 is expressed by neurons of the dorsal horn in normal conditions. After PSL, EZH2 expression is increased in neurons of the spinal cord dorsal horn in lesioned rats with neuropathic pain (Yadav and Weng, 2017). Besides, the number of microglia with EZH2 expression is drastically increased by more than sevenfold (Yadav and Weng, 2017). Inhibition of EZH2 attenuates the expression of inflammatory mediators (**Table 2**) and the development and maintenance of mechanical and thermal hyperalgesia in rats with PSL. In agreement with this study, Arifuzzaman et al. (2017) described that the use of EPZ-6438, an EZH2 inhibitor, dose-dependently inhibits LPS induced inflammatory gene expression in microglial cells *in vitro*. Genes found to be decreased by EZH2 inhibition after LPS activation were linked to cytokines, chemokines, enzymes, and transcription factors (Arifuzzaman et al., 2017). Thus, targeting the EZH2 signaling pathway could be an effective approach for the management of neuropathic pain, through immunomodulation.

**Table 2:** Epigenetic enzymes related to inflammation after injury.

Enzyme	Alteration after injury	Molecular effect	Effect on gene expression	Genes altered	Direct relation to pain	Reference
EZH2	Increased	Di- and tri-methylation of H3K27	Gene silencing	Indirect increase of TNF-α, IL-1β, and MCP-1	Yes	Arifuzzaman et al., 2017; Yadav and Weng, 2017
JMJD3	Increased	Demethylation of H3K27me2/3	De-repression	IL-6, BDNF	No	Lee et al., 2012; Palomer et al., 2016
MeCP2	Increased	Binding to CpG	Gene silencing	Indirect increase of IL-6, TNF-α, CXCL2, CXCL3, and CSF3	Yes	Wang et al., 2011; Tochiki et al., 2012; Cronk et al., 2015
DNMT3B	Decreased	CpG methylation	Gene silencing	CXC3R3	Yes	Jiang et al., 2017
HATs	Increased	H3K9 acetylation	Gene expression	CCL2 (MCP-1), CCL3 (MIP-1a), MiP-2, VEGFA, CXCR2, CXCR1/CXRR5, VEGFR, BDNF, COX2	Yes	Kiguchi et al., 2012, 2013, 2014; Sun et al., 2013; Zhu et al., 2014
HDAC1	Increased	H3K9 hypoacetylation	Reduction of gene expression		Yes	Kami et al., 2016
SIRT1	Decreased	H3 deacetylation	Reduction of gene expression	IL-6, INF-γ, IL-1β, TNF-α, and nuclear factor-kappa B (NF-κB) p65 activation	Yes	Yin et al., 2013; He et al., 2014; Shao et al., 2014; Gui et al., 2015
SIRT2	Decreased	H3 deacetylation	Reduction of gene expression	TNF-α, IL-1β, and IL-6, and acetylation of the NF-κB p65	Yes	Zhang and Chi, 2017
BET proteins	Unkown	Binding to acetylated histones	Gene expression	Cytokines, chemokines	No	Huang et al., 2009; Nicodeme et al., 2010; Gallagher et al., 2014

#### JMJD3

The histone demethylase that specifically demethylates H3K27me2/3 producing de-repression, JMJD3, seems also to be involved in inflammatory mechanisms which contribute to the physiopathology after CNS injury. For example, it has been described that spinal cord injury (SCI) produces an increase of JMJD3 in endothelial cells, inducing an increased expression of the cytokine IL-6 by demethylating its promoter (Lee et al., 2012). This event has been corroborated *in vitro*, JMJD3 siRNA inhibits the expression of IL-6 in response to oxygen/glucose deprivation (Lee et al., 2012). The acutely expressed IL-6 from endothelial cells after injury, may play a central role in early processes after a CNS lesion, since it influences the BBB integrity (Brett et al., 1995; Tinsley et al., 2009). Thus, JMJD3 is certainly related with the acute processes after a lesion on the CNS. However, a direct contribution of IL-6-induced by JMJD3 to neuropathic mechanisms, has still not been proven.

Further studies *in vitro* confirm the relation of JMJD3 on inflammation. JMJD3 expression increases after inflammatory stimuli such as LPS, and has been found to activate the expression of genes associated with inflammation in microglial and macrophage cultures through transcriptional regulation of Stat1 and Stat 3 (Lee et al., 2014; Przanowski et al., 2014). Besides JMJD3 contribution to inflammatory processes, can be also related through modulation of the expression of BDNF in DRG neurons after nerve lesions. BDNF has been found to increase in DRG after peripheral nerve injury, contributing to neuropathic pain. Thermal hyperalgesia and mechanical allodynia are inhibited with an antibody against BDNF administered intrathecally (Uchida et al., 2013). Usually, BDNF gene is silenced by PRC2, which contains as a catalytic subunit EZH2. After neuronal stimulation with NMDA *in vitro*, JMJD3 is recruited to the BDNF promoter, inducing demethylation of BDNF promoter. Thus, the de-repression of the promoter contributes to increase BDNF expression in mature neurons (Palomer et al., 2016). Besides promoter de-repression, BDNF expression is also enhanced through acetylation mechanisms, through the action of the CREB kinase/CBP. Thus, the epigenetic modification of the BDNF promoter has a crucial role in neuronal activation *in vitro* and may contribute to BDNF increased levels observed after neuronal injury *in vitro*. JMJD3 might be a therapeutic target to reduce neuropathic pain after a neural traumatic lesion by decreasing inflammation and neurotrophic factor expression.

#### MeCP2

MeCP2, which belongs to a transcriptional repressor complex together with a subset of HDACs, has been related to neuropathic pain after several nerve injuries. Priming for MeCP2 binding to DNA is DNA methylation at CpG, implemented by DNMTs. Once MeCP2 binds to the DNA, produces gene silencing. MeCP2 it is highly expressed in neurons of the dorsal horn and DRG, but also, in a lower extent in oligodendrocytes and astrocytes within the spinal horn (Wang et al., 2011; Tochiki et al., 2012). Increased DNMT, MeCP2 and HDACs expression has been observed after different pain states, paralleled by changes in MeCP2 target genes (Wang et al., 2011; Tochiki et al., 2012; Pollema-Mays et al., 2014). The neuropathic pain observed is markedly attenuated by the DNMT inhibitor 5-AZA treatment (Wang et al., 2011), which reduces DNA methylation and reactivate silenced genes (Holliday and Ho, 2002). Thus 5-AZA may alleviate neuropathic pain by upregulating the expression of some DNA methylation-dependent anti-nociceptive genes in the lumbar spinal cord in CCI rats. Although in the study authored by Wang et al., the molecular mechanisms by which MeCP2 may promote neuropathic pain where not described, it may be in part by promoting inflammatory mechanisms. It has been observed that MeCP2 is an important epigenetic regulator of macrophage response to stimuli and stressors (**Table 2**) (Cronk et al., 2015). Also, MeCP2 has been observed to be important for the expression of the opioid receptor, which will be commented later on.

However, controversial studies show that MeCP2 is decreased in the lumbar spinal cord, in DRG (Tochiki et al., 2012), and in the prefrontal cortex and amygdala (Tajerian et al., 2013) of mice after SNI, a lesion that produces persistent pain. These levels are reversed by environmental enrichment and correlated with decreased levels of hyperalgesia (Tajerian et al., 2013). These discrepancies may be related to the time window studied or methodologies used. In any case, further studies should be performed analyzing the DNA methylation and MeCP2 binding on specific genes related to nociception (reviewed in Geranton and Tochiki, 2015). Similarly, it has also been described that spinal nerve ligation (SNL) downregulates the expression of DNMT3b, which may cause demethylation of *C-X-C motif chemokine receptor 3 (Cxcr3)* gene promoter and increase CXCR3 expression in spinal neurons. CXCR3 is a receptor for the chemokine CXCL10, and binding of this chemokine facilitates excitatory synaptic transmission and contribute to the maintenance of neuropathic pain. The upregulated CXCR3 may contribute to neuropathic pain by facilitating central sensitization (Jiang et al., 2017).

Thus, literature has a discrepancy about the role of MeCP2 and DNMTs in neuropathic pain after traumatic injuries. Giving the importance of these events, further studies should be performed to clarify the molecular events underlying these epigenetic alterations.

### Histone Acetylation

Several studies suggest that modifications in histone tails (H3 and H4), acetylation and methylation, produce the transcription of inflammatory molecules, such as cytokines and chemokines, being the reason of chronic inflammatory diseases. In these case, HATs seem to be related to the chemokine expression, whereas HDACs are related to cytokine expression.

#### Histone Acetyltransferases

Nerve injury induces increased expression of chemokines and their receptors in infiltrated macrophages and neutrophils on the lesioned nerve, leading to neuropathic pain (**Table 2**). The induced expression of these proteins is concomitant with an increased H3K9Ac and tri-methylation of H3K4 (H3K4me3) and on their promoters (Kiguchi et al., 2012, 2013, 2014). Several studies demonstrated that the increased expression of CCL2, CCL3, MiP-2, CXCR2, and CXCR1/CXRR5 were suppressed by the HAT inhibitor anacardic acid, suggesting that these chemokines are upregulated through histone acetylation of H3K9. Moreover, this treatment also decreased the neuropathic pain associated to the nerve injury. Furthermore, another study observed an increased expression of CXCR2 and CCL1 by H3K9Ac in the spinal cord, being responsible of neuropathic pain induced after injury. Blocking CXCR2 reverses mechanical hypersensitivity after lesion (Sun et al., 2013). In agreement with this, treatment with suberoylanilide hydroxamic acid (a HDAC inhibitor) significantly exacerbated mechanical sensitization after incision (Sun et al., 2013). Similarly, Curcumin, which has been recognized as a p300/CBP inhibitor of the HAT activity, has been observed to have an anti-nociceptive role in the CCI rat model of neuropathic pain, through down-regulating p300/CBP HAT activity-mediated gene expression of BDNF and COX2 (Zhu et al., 2014). Thus, inhibition of HAT activity has been proven to reduce inflammation and neuropathic pain.

#### Histone Deacetylases

Recent studies have shown that HDAC inhibitors can alleviate inflammatory pain (Chiechio et al., 2009; Bai et al., 2010; Zhang et al., 2011) and attenuate the development of hypersensitivity in models of neuropathic pain (Zhang et al., 2011; Denk et al., 2013; Kukkar et al., 2014; Capasso et al., 2015). Since, HDACs inhibitors have demonstrated suppression of cytokine expression (Leoni et al., 2005; Kukkar et al., 2014; Khangura et al., 2017), decreased neuropathic pain through HDAC inhibitors may be related to suppression of inflammation through pro-inflammatory cytokine suppression.

Searching for specific HDACs, it has been described that neuropathic pain maintenance involves HDAC1, since the use of a HDAC1 specific inhibitor (LG325) dose-dependently ameliorated mechanical allodynia of SNI mice. Nerve injury increases HDAC1 as well as hypoacetylation of H3K9 within microglia of the dorsal horn (Kami et al., 2016). Furthermore, running exercise is to reduce HDAC1, increase H3K9Ac and reverse hyperalgesia in the mice (Kami et al., 2016). In another study, Baicalin, a natural compound, administration reversed H3 and HDAC1 expression in spinal cord, paralleled by a decrease of neuropathic pain after SNL (Cherng et al., 2014). Conversely, the HDAC1-HDAC6 inhibitor LG322, showed a less favorable antinociceptive profile (Sanna et al., 2017).

The histone deacetylase SIRT1 has a special interest. It has been observed that SIRT1 is decreased in the L4/L5 segments of the spinal cord after CCI, (Gui et al., 2015). Natural compounds that attenuate neuropathic pain, increase its expression within the spinal cord as well as decrease acetyl-histone H3 (Yin et al., 2013; He et al., 2014; Gui et al., 2015), reduce microglia activation, and expression of inflammatory modulators (**Table 2**) (Tillu et al., 2012; Gui et al., 2015; Wang et al., 2016). Reversion of mechanical and thermal hyperalgesia was observed with an specific SIRT1 inhibitor (Shao et al., 2014). Similarly, SIRT2 is downregulated in the DRG after CCI in rats. Overexpression of SIRT2 markedly alleviates mechanical allodynia and thermal hyperalgesia in CCI rats associated with inhibition of NF-κB signaling and inflammation (**Table 2**). Additionally, treatment with a SIRT2 specific inhibitor significantly aggravated neuropathic pain and attenuated the inhibitory effect of SIRT2 overexpression on neuropathic pain development. Therefore, SIRT2 and SIRT 1 may serve as potential therapeutic targets for treatment of neuropathic pain (Zhang and Chi, 2017).

#### BET Proteins

The BET protein family is comprised of BRD2, BRD3, BRD4 and BRDT, and regulates RNA Polymerase II (Pol II)-dependent gene expression by recruiting transcriptional regulatory complexes to poly-acetylated chromatin. Recently, BET proteins have been intensively studied as epigenetic regulators of cell cycle and inflammation in many disorders such as cancer and arthritis. However, virtually nothing is known about the role of BET proteins during the injury response after CNS trauma. It has been described that BET inhibition reduces the expression of pro-inflammatory cytokines/chemokines in macrophages after LPS stimulation (Nicodeme et al., 2010). In several contexts, NF-κB-mediated inflammatory signaling is disrupted after BET inhibition treatment (Huang et al., 2009; Gallagher et al., 2014). Therefore, there is a clear link between BET proteins and NF-κB-mediated inflammatory signaling, which can be targeted for therapeutic purposes. Thus, the BET proteins have an important role in inflammation and cell proliferation. However, its implication in neuropathic pain remains unknown.

### Non-coding RNAs

Most of the studied non-coding RNAs related to pain are microRNAs (**Table 3**). For example, diverse microRNAs directly regulate the levels of the regulator of inflammation named suppressor of cytokine signaling 1 (SOCS1), and thus control neuropathic pain by modulating inflammation. SOCS1 mRNA is a direct target of miR-221, miR-155 and miR-19a. After CCI these microRNAs increase, producing decreased levels of SOCS1. Inhibition of these microRNAs abrogate SOCS1 depletion, and mechanical and thermal hyperalgesia (Tan et al., 2015; Wang et al., 2015; Xia et al., 2016). Similarly, downregulation of microRNA-218 by a specific inhibitor relieves neuropathic pain by regulating suppressor of cytokine signaling 3 (SOCS3) after CCI in rats (Li and Zhao, 2016).

**Table 3:** miRNAs related to inflammation and neuropathic pain after traumatic injury.

miRNA	Expression after injury	Site	Genes altered	Reference
miR-221, miR-155, miR-19a	Increased	Microglia	SOCS1	Tan et al., 2015; Wang et al., 2015; Xia et al., 2016
miR-221	Increased	Microglia	TNF-α, IL-1β, IL-6, NF-κB, p38 MAPK activation	Xia et al., 2016
microRNA-218	Increased	Microglia	SOCS3	Li and Zhao, 2016
miR-206	Increased	DRG	BDNF	Neumann et al., 2015
miR-1	Increased	Sciatic nerve	BDNF	Sun W. et al., 2017
miR-21	Increased	DRG	Unknown	Sakai and Suzuki, 2013
miR-32-5p	Increased	Microglia	Dusp5	Yan et al., 2018
miR-195	Increased	Microglia	Indirect effect on IL-1β, TNF-α, iNOS	Shi et al., 2013
miR-124	Decreased	Microglia	Unknown	Willemen et al., 2012
MicroRNA-146a-5p	Decreased	Spinal cord	TRAF6, JNK/CCL2	Lu et al., 2015
miR-93	Decreased	Spinal cord	STAT3	Yan et al., 2017

The levels of BDNF are also important in neuropathic pain formation and maintenance, and are linked to microRNA expression. On one side, miRNA microarray analysis reveals that 167 miRNAs are altered in DRG after following BDNF gene deletion (Neumann et al., 2016). On the other side, microRNAs tightly regulate the levels of BDNF. BDNF is controlled by miR-206 in DRG (Neumann et al., 2015), and by miR-1 in sciatic nerve (Sun W. et al., 2017), following CCI. Both microRNAs decrease after injury and induce BDNF expression, producing mechanical and thermal hyperalgesia. Inhibition of miR-206, which directly interacts at the 3′-UTR of BDNF, also decreases pro-inflammatory cytokine expression and neuropathic pain development (Sun W. et al., 2017).

Besides, there are several others upregulated microRNAs that regulate neuropathic pain and inflammation. Nerve injury induces upregulated expression of miR-21 in DRG neurons (Sakai and Suzuki, 2013), and miR-32-5p (Yan et al., 2018) and miR-195 (Shi et al., 2013) in spinal microglia. Investigations related to the increased miR-32-5p in spinal microglia after SNL, revealed Dual-specificity phosphatase 5 (Dusp5) as a direct target of this microRNA, which is involved in mediating the effects on neuropathic pain and neuroinflammation (Yan et al., 2018). Interestingly, miR-195 aggravates inflammation and neuropathic pain by inhibiting autophagy after SNL (Shi et al., 2013).

Finally, it is important to mention that not all the microRNAs have a pathogenic effect. In fact, injury induces the reduction of several microRNAs, and intrathecal delivery of some of them, may be a therapeutically effective on reducing pain symptoms. miR-124 levels are associated with M1/M2 microglia phenotype, and inflammatory signaling. Intrathecal miR-124 treatment reverses the persistent hyperalgesia induced and mechanical allodynia several models of chronic neuropathic pain in mice (Willemen et al., 2012). miRNA-146a-5p attenuates neuropathic pain via suppressing TNF receptor associated factor-6 (TRAF6) and its downstream signaling JNK/CCL2 in the spinal cord (Lu et al., 2015). There is also decreased miR-93 in the spinal cord of CCI rats compared with sham rats, which directly targets the signal transducer and activator of transcription 3 (STAT3), an important regulator of inflammation. Overexpression of miR-93 markedly suppresses the expression of STAT3 *in vitro* and *in vivo*, and significantly alleviates inflammation and neuropathic pain development in CCI rats (Yan et al., 2017).

## Altered Gene Expression Related to Neuronal Hyperexcitability

Traumatic injuries produce neuronal hyperexcitability. Although the exact mechanisms vary depending on site and pathology, peripheral and central sensitization lead to the development of neuropathic pain (reviewed in Cohen and Mao, 2014; Ligon et al., 2016). After nerve injury, the pain pathway involves the activation of primary nociceptive afferents within the periphery, which then send impulses to the dorsal horn of the spinal cord, where second order nociceptive neurons convey ascending signals to the thalamus, amygdala, and the brain cortex. If the injury occurs in the spinal cord, signals descend to nociceptors at the dorsal horn, as well as ascend to supraspinal regions. These lesions produce also the loss of inhibitory control from supraspinal regions onto primary afferent neurons, which contributes to exacerbate pain. In any case, neuronal sensitization is promoted through changes in gene regulation that shift the balance of channels, receptors, neurotransmitters or transporters, leading to an increased excitably of the neuron. Most of these gene expression alterations are produced by changes in histone or DNA methylation in their promoters, although some changes in promoter acetylation are also described. In particular, it has been clearly demonstrated that injury in the nervous system triggers an increase of promoter methylation producing gene silencing.

### Histone and DNA Methylation

**FIGURE 2:**
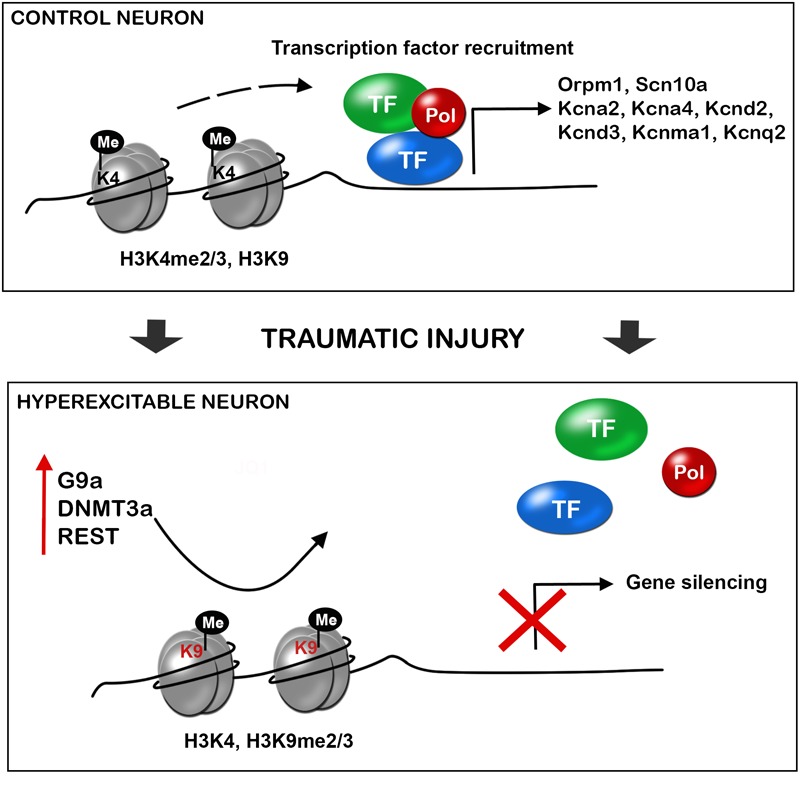
Traumatic injuries of the nervous system induce gene expression alteration in neurons triggering hyperexcitability and neuropathic pain. During control conditions, the promoter state of genes codifying for ion channels, receptors and other important neuronal genes allow the recruitment of transcription factors, and thus gene expression. After a traumatic injury of the nervous system, there is an increased expression of methyltransferases (G9a, histone-lysine *N*-methyltransferase; DNMT3a, DNA methyltransferase a), and the repressor complex REST (RE1-silencing transcription factor), with decrease H3K4 methylation and increase H3K9 methylation. This alteration of the promoter state does not allow transcription factor recruitment and there is silencing of several neuronal genes. De-balanced expression of ion channel, receptors, transporters, and other neuronal genes triggers neuronal hyperexcitability and consequent neuropathic pain.

Nerve injury produces the upregulation of the methyltransferases such as EHMT2 or G9a, DNA methyltransferase 3 alpha (DNMT3a), and the repressive complex RE1-Silencing Transcription factor (REST, also known as Neuron-Restrictive Silencer Factor, NRSF), that produce gene silencing. There is a clear effect of these over-expressed epigenetic enzymes on the silencing of similar target genes (**Figure 2**). Besides, several microRNAs have been described to alter neuronal excitability after neural injury.

#### G9a

G9a (encoded by Ehmt2), is a H3K9 methyltransferase, responsible of gene silencing and it has been clearly demonstrated that it contributes to transcriptional repression in primary sensory neurons contributing to neuropathic pain. Nerve injury consistently increases the enrichment of H3K9me2 in the promoters of potassium channels, producing their silencing. A study demonstrated that the specific knockout of G9a in sensory neurons of the DRG blocked gene silencing of potassium channels (**Table 4**) and the promotion of neuropathic pain after peripheral nerve lesion (Laumet et al., 2015).

**Table 4:** Epigenetic enzymes related to neuronal hyperexcitability after injury.

Enzyme	Expression after injury	Molecular effect	Effect on gene expression	Genes altered	Direct relation to pain	Reference
G9a	Increased	H3K9 methyltransferase	Gene silencing	Kcna4, Kcnd2, Kcnq2 and Kcnma1, Orpm1	Yes	Zhou et al., 2014; Zhang et al., 2016
DNMT3a	Increased	CpG methyltransferase	Gene silencing	Orpm1 (MOR), Oprk1 (KOR), channel expression as Kcna2	Yes	Shao et al., 2017; Sun L. et al., 2017; Zhao et al., 2017
REST	Increased	H3K4 demethylation and histone deacetylation	Gene silencing	Kcnd3, Kcnq2 and Scn10a, Oprm1 and Gad2	Yes	Uchida et al., 2010a; Zhang et al., 2011
HDAC	Increased	H3 and H4 deacetylation	Gene silencing	Gad65, mGlu2, MOR, DOR and Nav1.8	Yes	Chiechio et al., 2006, 2010; Uchida et al., 2010a; Zhang et al., 2011; Matsushita et al., 2013; Zammataro et al., 2014; Tao et al., 2016; Hou et al., 2017

Other studies demonstrated that targeting G9a reverses the silencing of the Orpm1 gene, which encodes the MOR, and restores the effect of morphine on the hypersensitivity induced by peripheral nerve lesions (Zhou et al., 2014; Zhang et al., 2016). Opioids are the gold standard for pharmacological treatment of pain, but their analgesic effects are unsatisfactory in conditions of neuropathic pain, due in part to nerve injury–induced downregulation of opioid receptors in DRG and spinal neurons. Nerve lesion produces a decreased expression of MOR in DRG, by increasing H3K9me2 in its promoter, reducing the opioid effect on neuropathic pain (Zhou et al., 2014; Zhang et al., 2016). The knockout of G9a reverses the expression of MOR in lesioned DRG and potentiates the effect of morphine after lesion (Zhang et al., 2016). Similarly, treatment with the inhibitor of DNA methylation 5-aza-dC, inhibited the increased methylation of MOR gene and prevented their decreased expression in DRG, thereby improving systemic, spinal and peripheral morphine analgesia (Zhou et al., 2014).

#### DNA Methyltransferases

Similar to the histone methyltransferase G9a, the DNA methyltransferases DNMT3a has been found to have similar effects on MOR and ionic channel expression. Nerve injury induces increased expression of DNMT3a in lesioned DRG neurons and represses expression of Orpm1 and Oprk1 genes which encode for MOR and KOR (Shao et al., 2017; Sun L. et al., 2017). Blocking this increase with a DNMT3a shRNA-adenoviral vector, restored MOR and KOR expression as well as restored morphine analgesic effects. Mechanistically, DNMT3a regulation of Oprm1 gene expression required the methyl-CpG–binding protein 1, MBD1, as MBD1 knockout resulted in the decreased binding of DNMT3a to the Oprm1 gene promoter and blocked the DNMT3a-triggered repression of Oprm1 gene expression in DRG neurons (Sun L. et al., 2017).

Besides, increased DNMT3a expression in the injured DRG neurons by SNL promotes the decrease of the voltage-dependent potassium channel subunit Kcna2. Blocking this increase with a DNMT3a shRNA-adenoviral vector, prevents nerve injury-induced methylation of the subunit Kcna2 promoter region, rescues Kcna2 expression in the injured DRG and attenuates neuropathic pain (Zhao et al., 2017). In agreement with this observation, in the absence of nerve injury, mimicking the increase of DNMT3a reduces the Kcna2 promoter activity, diminishes Kcna2 expression, decreases potassium current, increases excitability in DRG neurons and leads to spinal cord central sensitization and neuropathic pain symptoms (Zhao et al., 2017). These findings suggest that DNMT3a may contribute to neuropathic pain by repressing MOR, KOR and Kcna2 expression in the DRG (Zhao et al., 2017). Consistently, DNA methylation by DNMTs alters voltage gated and Ca2+ ion channels, affecting neuronal excitability (Meadows et al., 2016).

#### REST

The enzymatic core of the REST repressor complex contains REST, CoREST, LSD1, BHC80, and BRAF35 (Mosammaparast and Shi, 2010). The REST complex also recruits additional silencing molecular machineries, such as HDAC1/2, MeCP2 and G9a, to consolidate the suppression (Ballas et al., 2005). The REST complex suppresses gene expression by removing active histone marks, such as H3K4 methylation or various histone acetylation. REST suppresses the expression of genes important for the acquisition and preservation of neuronal specificity. Thus, in the adult brain, neurons exhibit low levels of REST (Gao et al., 2011). The decrease of REST expression during neuronal differentiation, allows the expression of genes that activate a large variety of processes such as axonal growth, establishment of synaptic contacts and membrane excitability (Paquette et al., 2000; Aoki et al., 2012). Conversely, the levels of REST are high in the majority of non-neuronal cells (Prada et al., 2011).

In several pathologic conditions, REST is induced in neurons, which is associated with the repression of specific neuronal genes (Baldelli and Meldolesi, 2015). This increase of REST expression has been observed in the CNS after ischemia, epileptic seizures, as well as in the PNS after nerve lesions, and *in vitro* after prolonged neuronal depolarization (Hara et al., 2009; Uchida et al., 2010a; Roopra et al., 2012; Schweizer et al., 2013; Bersten et al., 2014; Baldelli and Meldolesi, 2015). If these increases of REST are protective or detrimental is still controversial. However, it is clearly demonstrated that neuropathic pain is associated with REST overexpression in the PNS.

Studies using the PNL of the sciatic nerve in the mouse, showed an increase of the REST mRNA and protein in DRG, which is maintained during weeks after lesion (Uchida et al., 2010a,b; Rose et al., 2011). The expression levels of target genes for REST such as Kcnd3, Kcnq2 and Scn10a (that respectively encode for the channels Kv4.3, Kv7.2 and Nav1.8), as well as Oprm1 and Gad2 are repressed in response to the increased levels of REST (**Table 4**). The repression of these target genes is associated with persistent dysfunction of nociceptive C-fibers and disruption of H and M currents, that facilitate the neuropathic excitability of the peripheral sensory fibers. Thus, increased REST expression enhances the generation and maintenance of neuropathic pain. REST knockdown with antisense nucleotides is sufficient to rescue the expression levels of several target genes, as well as C-nociceptive fiber function (Uchida et al., 2010a; Zhang et al., 2011).

### Acetylation

Finally, some studies indicate that HDAC inhibition or acetylation in histones in neuropathic pain models have analgesic effects. Most of these studies have their target in inflammation, as they have been explained in a previous section. However, other studies have demonstrated that HDAC inhibition also affects channel expression in neurons. For example, induced inflammation produces a decrease of acetilated-H3 in *Gad65* promoter, decreasing the expression of GAD65. GAD65 is an essential enzyme for GABAergic neuron function in the dorsal horn of the spinal cord and in the raphe nucleus in the brain. Administration of TSA or SAHA restores *Gad65* acetylation and relieve sensitive pain behavior in raphe nucleus (Zhang et al., 2011).

Another analgesic effect of HDAC inhibitors is through increasing metabotropic glutamate 2 receptors (mGLU2). mGLU2 receptors are at the synapses between primary afferent fibers and neurons in the dorsal horn of the spinal cord, in the peripheral nociceptors, and in pain-regulatory centers of the brainstem and forebrain. Activation of these receptors inhibits pain transmission. HDAC inhibitors transcriptionally increase mGLU receptors via the acetylation-promoted activation of the p65/RELA transcription factor (Chiechio et al., 2006, 2010). Thus, HDAC inhibitors increase acetylated p65 in the DRG and spinal cord dorsal horn, which regulates p65 retention in the nucleus, increasing NF-κB function on mGLU2 transcriptional activation (Chiechio et al., 2006, 2009) after CCI or inflammatory-induced pain. Induction of analgesia is inhibited by the mGLU2/3 receptor antagonist, LY341495 (Chiechio et al., 2002; Descalzi et al., 2015). Moreover, the analgesic effect of a mGLU2/3 agonist was enhanced after HDAC inhibitor treatment (SAHA) (Zammataro et al., 2014).

Additional evidence for the importance of histone acetylation has been related to MOR, DORs and Nav1.8 expression. The expression of these receptors and channels is reduced under neuropathic pain states (Uchida et al., 2010a; Tao et al., 2016), produced by a reduction of acetylation in its promoters. Treatment with HDAC inhibitors, such as TSA, VPA and SAHA treatments, that increase acetylation at the regulatory sequence of these genes, promotes C-fiber related hypoesthesia and restores peripheral and systemic morphine analgesia (Matsushita et al., 2013; Uchida et al., 2015; Hou et al., 2017). These results suggest that HDAC inhibitors could serve as adjuvant analgesics to morphine for the management of neuropathic pain.

### Non-coding RNAs

Several miRNas have been observed to change their expression in models of neuropathic pain. For instance, miR-7a is the most robustly decreased microRNA in the injured DRG, and is associated to neuropathic pain through regulation of neuronal excitability. Overexpression of this microRNA, suppresses established neuropathic pain, and downregulation is sufficient to cause pain-related behaviors in intact rats. miR-7a targets b2 subunit of the voltage-gated sodium channel. Thus, after injury decreased miR-7a allows b2 subunit protein expression, triggering hyperexcitability of nociceptive neurons (Sakai et al., 2013). Sodium voltage gated channels have been observed to me regulated by other miRNAs. For example, miRN-96 inhibits Nav1.3 expression and alleviates neuropathic pain after CCI. Injury increases Nav1.3 expression and intrathecal administration of miR-96 suppresses this expression (Chen et al., 2014). Similarly, miR-30b controls the expression of Nav1.7 after SNI. miR-30b over-expression in spared nerve injury rats inhibits SCN9A transcription, resulting in pain relief. In addition, miR-30b knockdown significantly increased hypersensitivity to pain in naive rats (Shao et al., 2016).

It has been established also, that the cluster of microRNAs that includes miR-96, -182, and -183, has a reduced expression in primary afferent neurons DRG in a model of SNL. The redistribution of microRNAs is associated with altered distribution of the stress granule protein TIA-1, which may have a significant impact on regulatory activity of microRNAs (Bhattacharyya et al., 2006; Vasudevan and Steitz, 2007; Aldrich et al., 2009). Specifically, the microRNA-183 cluster in mice controls more than 80% of neuropathic pain–regulated genes and scales basal mechanical sensitivity and mechanical allodynia. For example, controls voltage-gated calcium channel subunits α2δ-1 and α2δ-2 and TrkB+ light-touch mechanoreceptors (Peng et al., 2017). Besides, another microRNA that controls calcium voltage-gated channels is miR-103, which regulates the expression of the three subunits forming Cav1.2-comprising L-type calcium channel (LTC). This regulation is bidirectional since knocking-down or over-expressing miR-103, respectively, up- or down-regulate the level of Cav1.2-LTC translation. Besides, miR-103 knockdown in intact rats results in hypersensitivity to pain. This miRNA, is downregulated in neuropathic pain animals, and its intrathechal administration relieve pain after SNL (Favereaux et al., 2011).

## Conclusion

Epigenetic alterations produced after injury of the CNS or PNS contribute to the generation and maintenance of neuropathic pain. Current literature describes clear effects of DNA methylation, histone methylation and acetylation, and microRNAs on the expression of ion channels, receptors and neurotransmitters in neurons. HATs affect chemokine expression whereas HDACs affect cytokine expression within glial and macrophage cells that are reactive to neuronal damage. However, there is no agreement regarding histone and DNA methylation effects on inflammatory mechanisms that sustain pain states. Although increasing interest is shown within this epigenetic field, we are still at the initial steps of understanding these processes. Thus, further research needs to be performed to evaluate novel therapies that might be effective on patients that suffer from neuropathic conditions.

## Author Contributions

CP and XN reviewed the bibliography, wrote the text, and drew the figures of the present review.

## Conflict of Interest Statement

The authors declare that the research was conducted in the absence of any commercial or financial relationships that could be construed as a potential conflict of interest.

## Abbreviations

5-AZA: 5-azacytidine
5-aza-dC: 5-aza-2′-deoxycytidine
BBB: blood-brain barrier
BDNF: brain-derived neurotrophic factor
BET: bromodomain and extra terminal domain
CBP: CREB-binding protein
CCI: chronic constriction injury
CCL/MCP: C-C motif chemokine ligand/monocyte chemoattractant protein
CNS: central nervous system
COX2: cyclooxygenase-2
CREP: cyclic AMP response element-binding protein
CXCR: C-X-C motif chemokine receptor
DNMTs: DNA methyltransferases
DOR: δ-opioid receptor
DRG: dorsal root ganglia
EHMT2 or G9a: euchromatic histone-lysine *N*-methyltransferase
EZH2: enhancer of zeste 2 polycomb repressive complex 2 subunit
H3K27me2/3: histone 3 lysine 27 di and trimethylation
H3K4me3: histone 3 lysine 4 trimethylation
H3K4me3: trimetilation H3K4
H3K9Ac: H3K9 acetylation
H3K9Ac: histone 3 lysine 9 acetylation
HATs: histone acetyltransferase enzymes
HDACs: histone deacetylases
JMJD3 or KDM6b: jumonji domain containing 3
KOR: κ-opioid receptor
MeCP2: methyl-CpG-binding protein 2
mGLU: metabotropic glutamate receptor
MOR: μ-opioid receptor
NF-κB: nuclear factor-kappa B
NGF: nerve growth factor
NMDA: *N*-methyl-D-aspartate
Orpm1: opioid mu receptor gene
PNS: peripheral nervous system
PRC2: polycomb repressive complex 2
PSL: partial sciatic nerve ligation
(REST, also known as Neuron-Restrictive Silencer Factor, NRSF): RE1-Silencing Transcription factor
SAHA: voronistat
SIRT: sirtuin
TSA: trichostatin A
VEGF: vascular endothelial growth factor
